# A Combined Approach for the Assessment of Cell Viability and Cell Functionality of Human Fibrochondrocytes for Use in Tissue Engineering

**DOI:** 10.1371/journal.pone.0051961

**Published:** 2012-12-18

**Authors:** Ingrid Garzón, Victor Carriel, Ana Belén Marín-Fernández, Ana Celeste Oliveira, Juan Garrido-Gómez, Antonio Campos, María del Carmen Sánchez-Quevedo, Miguel Alaminos

**Affiliations:** 1 Department of Histology (Tissue Engineering Group), University of Granada, Granada, Spain; 2 Division of Oral and Maxillofacial surgery, University Hospital Virgen de las Nieves, Granada, Spain; 3 Division of Trauma and Orthopedic Surgery, University Hospital San Cecilio, Granada, Spain; University of Minho, Portugal

## Abstract

Temporo-mandibular joint disc disorders are highly prevalent in adult populations. Autologous chondrocyte implantation is a well-established method for the treatment of several chondral defects. However, very few studies have been carried out using human fibrous chondrocytes from the temporo-mandibular joint (TMJ). One of the main drawbacks associated to chondrocyte cell culture is the possibility that chondrocyte cells kept in culture tend to de-differentiate and to lose cell viability under in in-vitro conditions. In this work, we have isolated human temporo-mandibular joint fibrochondrocytes (TMJF) from human disc and we have used a highly-sensitive technique to determine cell viability, cell proliferation and gene expression of nine consecutive cell passages to determine the most appropriate cell passage for use in tissue engineering and future clinical use. Our results revealed that the most potentially viable and functional cell passages were P5–P6, in which an adequate equilibrium between cell viability and the capability to synthesize all major extracellular matrix components exists. The combined action of pro-apoptotic (TRAF5, PHLDA1) and anti-apoptotic genes (SON, HTT, FAIM2) may explain the differential cell viability levels that we found in this study. These results suggest that TMJF should be used at P5–P6 for cell therapy protocols.

## Introduction

The temporo-mandibular joint (TMJ) disc is a fibrocartilaginous tissue that lies between the mandibular condyle and the temporal fossa-eminence. Several disorders may affect the TMJ disc, including intra-articular positional and structural abnormalities with high prevalence in adult populations, especially TMJ degenerative diseases, known as osteoarthrosis or osteoarthritis. Clinical management of the most prevalent TMJ disc disorders is very challenging due to the low regeneration capability of human cartilage, and emerging therapies based on cultured human TMJF and tissue engineering represent a novel treatment possibility [1], [2].

The TMJ disc is mainly composed by fibrochondrocytes (TMJF), which have features of both chondrocytes and fibroblasts [3]. Human TMJF are known to have the capability to synthetize different fibrillar extracellular matrix (ECM) constituents, mainly collagen, and several non-fibrillar components, and to proliferate faster than hyaline chondrocytes [4]. The distribution of TMJF into the disc appears to be heterogeneous, and cells tend to show a round morphology surrounded by pericellular matrix.

Several efforts are currently ongoing in the field of TMJ disc tissue engineering using an immense variety of scaffolds and cell sources [5], [6], [7]. Nevertheless, the scarce number of cells that can be obtained from small TMJ disc tissue biopsies and the drop of cell viability and cell differentiation levels caused by continuous cell passaging in order to obtain large amounts of cells, are significant limitations associated to TMJF culturing and TMJ disc tissue engineering [8], [9]. All these limitations can result in the failure of cell therapy and tissue engineering strategies of the human TMJ disc repair. For these reasons, a deep study of sequential cell passages of cultured human TMJF might be a useful tool for tissue engineers in order to select the most suitable cell passage in terms of cell viability and differentiation from a clinical standpoint. In fact, several previous studies previously demonstrated that cell viability may vary among several cell passages and that selection of the most adequate cell passage is very important for cell therapy success [10], [11].

In this study, we carried out a comprehensive analysis of cell proliferation, cell viability and cell function on 9 consecutive cell passages of human TMJF to determine which passage is the most adequate for future clinical use.

## Materials and Methods

### Isolation and Seeding of TMJF

TMJF were isolated from the retrodiscal area of human adult TMJ discs. First, biopsies were obtained during arthroscopical examination in patients with temporo-mandibular dysfunction syndrome without involvement of the retrodiscal area. Samples were kept at 4°C in Dulbecco’s modified Eagle’s medium (DMEM; Sigma-Aldrich) supplemented with antibiotics and antimycotics (100 U/ml of penicillin G, 100 mg/ml of streptomycin and 0.25 mg/ml of amphotericin B; Sigma-Aldrich) and processed in the following 24 h. Then, an overnight enzymatic digestion was performed by using 2 mg/ml collagenase type II *Clostridium hystoliticum* (Gibco BRL Life Technologies Ref. 17100-017, Karlsruhe, Germany). Isolated cells were cultured on tissue culture flasks using a 3∶1 mixture of DMEM and Ham’s F12 culture media supplemented with 10% fetal bovine serum (FBS), 1% antibiotics, 24 µg/ml adenine, 0.4 µg/ml hydrocortisone, 5 µg/ml insulin, 10 ng/ml epidermal growth factor, 1.3 ng/ml triiodothyronine and 8 ng/ml of cholera toxin (all from Sigma-Aldrich). Subconfluent cells were passaged with 0.05% trypsin- EDTA (Sigma-Aldrich ref. T4299) and subcultured for nine consecutive passages (P1 to P9). All patients gave their written consent to participate in the study. This work was approved by the Research Ethics Committee of the Andalusian Public Health System (Comité de Ética de la Investigación del SSPA CEI-Granada).

### Determination of Cell Viability by Trypan Blue Dye Exclusion Test and LIVE/DEAD® Assay

To determine cell viability by using a dye exclusion test, we first used Trypan blue methods. After cell isolation, subconfluent cultures of TMJF were detached by using trypsin-EDTA for 8 min at 37°C and 100 µl of the cell suspension were stained and mixed with 0.4% trypan blue solution (Sigma-Aldrich ref. T8154) and incubated for 5 minutes at room temperature. The percentage of viable cells was quantified using a Neubauer chamber and a Nikon Eclipse 90 i light microscope by counting a minimum of 200 cells per cell passage. For each cell passage, a total of 6 determinations were carried out and means and standard deviations were calculated.

Then, we used a combined dye exclusion test (ethidium homodimer-1) and a cytoplasmic metabolic analysis (Calcein acetoxymethyl) to determine cell viability. With this purpose, we used Live/Dead™ Viability/Cytotoxicity kit (Molecular Probes, UK). Briefly, TMJF were seeded on chamber slides (Lab-Tek Chamber Slides, Nunc, Roskilde, Denmark) in a cell density of 5×10^3^, cells were washed with phosphate-buffered saline (PBS) for 30 min, and samples were then incubated in 200 µl a Live/Dead™ Viability/Cytotoxicity solution for 30 min. After staining, samples were washed and observed using a Nikon Eclipse 90i fluorescence microscope. For each cell passage, a total of 6 determinations were carried out and the mean and standard deviation was calculated.

### Determination of Cell Viability by Electron-probe X-ray Microanalysis (EPXMA)

For EPXMA, TMJF cells were cultured on pretreated pioloform-covered gold grids in a cell density of 5×10^3^ following previously described methods [12], [13]. Afterwards, gold grids containing TMJF were washed to remove extracellular medium using ice-cold distilled water for 5 sec and immediately drained and plunge-frozen in liquid nitrogen. After cryofixation, cells were freeze-dried for 23 h using a 6-segment protocol from −100°C to 25°C using a EMITECH K775X equipment. Subsequently, samples were carbon-coated. Electron probe X-ray microanalysis of the specimens was performed with a Philips XL30 scanning electron microscope (SEM) equipped with an EDAX DX-4 microanalytical system and a solid-state backscattered electron detector. Samples were examined with SEM with a combination of secondary electron (SE) and backscattered electron (BSE) imaging modes. For X-ray microanalysis, the analytical conditions were: tilt angle 0°, take-off angle 61.34° and working distance 10 mm. The acceleration voltage was 10 kV. All spectra were collected in the spot mode at 10,000× (equivalent to 50 nm spot diameter) for 200 sec live time, and the number of counts per second recorded by the detector was around 500. All determinations were performed on the central area of the cell nucleus. To determine total intracellular ionic content, we used the peak-to-local-background (P/B) ratio method [14] with reference to standards composed of 20% dextran containing known amounts of inorganic salts [15]. In this work, we analyzed the ionic content of 35 TMJF corresponding to each cell passage (P1 to P9) using 4 different individuals of each passage.

### Global Gene Expression Analysis by Gene Expression Microarray

Total RNA was isolated from two different samples corresponding to TMJF cells at each cell passage (P1 to P9) by using Qiagen RNeasy System™ (Qiagen, Mississauga, Ontario, Canada). Total RNA was converted into cDNA using a reverse transcriptase (Superscript II, Life Technologies, Inc., Carlsbad, California, EEUU) and a T7-oligo(dT) primer. Then, biotinilated cRNA was generated by using a T7 RNA polymerase and biotin-11-uridine-5′-triphosphate (Enzo Diagnostics, Farmingdale, Nueva York, EEUU). Labeled cRNA were chemically fragmented to facilitate the process of hybridization and hybridized to Affymetrix Human Genome U133 plus 2.0 oligonucleotide arrays for 6 hours at 45°C.

### Statistical Analysis

For trypan blue and Live/Dead™ Viability/Cytotoxicity methods, statistical differences between cell viability levels corresponding to two consecutive cell passages (for instance, P1 vs. P2), were analyzed by using Wilcoxon non-parametric tests. To compare the 9 cell passages globally (P1 to P9), we used Kendall W tests. The same tests were used to compare intracellular ionic contents of Na, K, Ca, Cl, Mg, P and S corresponding to 9 cell passages. All tests were carried out double-tailed, and p values below 0.01 were considered as statistically significant.

To determine the cell viability index for each cell passage, we first normalized the cell viability levels obtained for each particular method (trypan blue, Live/Dead™ Viability/Cytotoxicity and EPXMA K/Na ratio) to z-scores (mean = 0 and standard deviation = 1) using the formula: Z = (X-µ)/σ, where µ is the average cell viability obtained for each individual method, X is the specific cell viability for a specific cell passage, and σ is the standard deviation for each particular method. Then, a global average was calculated for each cell passage using the normalized values obtained from the three methods separately.

For the analysis of expression values as determined by microarray, we first selected three groups of genes and probe-sets by using the information provided by Affymetrix. These groups of genes were related to 1) cell viability, apoptosis and cell death; 2) extracellular matrix components; and 3) PCNA and MKI67 cell proliferation genes.

For the analysis of genes with significant correlation with the cell viability index, we used the 3.09 b version of the Significance Analysis of Microarrays (SAM) software of Stanford University, using a δ value that permitted a false discovery rate of 0 (i.e., no genes are falsely named). A multiclass analysis of all cell viability, apoptosis and cell death genes associated with the cell viability index was performed. This program is available at http://www-stat.stanford.edu/~tibs/SAM/.

To determine the correlation between sequential cell passaging and gene expression of the main ECM components, we used Pearson (r) correlation tests. All genes with a r correlation coefficient >0.700 were selected as positively correlated with cell passaging, whereas genes with a r correlation coefficient <−0.700 were selected as negatively correlated with cell passaging.

To compare PCNA and MKI67 gene expression levels between two consecutive cell passages (for instance, P1 vs. P2), we used Wilcoxon non-parametric tests. To analyze the 9 cell passages globally (P1 to P9), we used Kendall W tests. To determine a correlation between the expression of PCNA and MKI67, we used Kendal Tau correlation tests.

## Results

### Analysis of Cell Viability of 9 Consecutive Cell Passages of TMJF

First, trypan blue exclusion tests demonstrated that the cell viability of nine consecutive TMJF passages was very high, with more than 93% of viable cells at each cell passage, and with a maximum cell viability rate of 99.46% at P6 (Table 1). The statistical analysis (Table 2) demonstrated the existence of global significant differences among all 9 cell passages analyzed here (p = 0.000 for the W test of Kendall). However, pair-wise comparisons between two consecutive passages did not reveal any significant differences in cell survival among consecutive passages, although a non-significant trend to decrease was found until P4, with a subsequent increase to P6 and a final decrease until P9, with the lowest viability corresponding to P9.

**Table 1:** Analysis of cell viability and ionic content of 9 consecutive cell passages (P1 to P9) of TMJF.

	P1	P2	P3	P4	P5	P6	P7	P8	P9
**TRYPAN BLUE Exclusion test**	96.80±1.43	98.04±1.21	95.33±1.11	93.95±3.94	98.57±0.56	99.46±0.07	98.09±0.40	96.87±2.30	93.36±3.33
**LIVE/DEAD™ Cell Viability Assay**	91.29±5.51	89.65±8.16	95.39±4.68	95.02±2.14	96.29±1.88	96.77±1.48	97.38±1.66	94.70±2.31	93.59±2.68
**[Ca]**	10.05±8.86	9.62±7.02	14.27±12.38	13.03±8.64	7.28±7.18	8.35±11.32	7.56±10.93	4.83±5.49	10.28±9.49
**[Cl]**	137.70±29.70	144.81±47.26	95.64±31.81	115.31±40.88	123.25±30.58	165.92±50.21	111.19±48.16	126.87±43.61	132.34±50.44
**[K]**	253.02±78.90	182.50±45.40	167.79±65.77	279.50±93.11	253.82±66.38	303.73±91.13	189.92±64.17	311.74±107.78	203.77±67.85
**[Mg]**	21.39±5.94	19.38±5.33	18.34±6.05	25.75±7.89	22.62±6.67	20.99±5.84	16.09±4.11	30.05±8.98	23.22±8.60
**[Na]**	53.38±22.82	84.65±36.43	43.62±19.47	58.86±28.73	43.77±29.07	43.21±27.18	36.16±28.34	53.24±18.45	97.78±71.59
**[P]**	258.45±27.80	226.52±44.88	191.81±59.92	282.71±56.77	226.68±43.70	280.67±83.92	190.62±55.23	311.61±69.75	233.25±95.32
**[S]**	88.21±21.94	64.78±10.36	58.46±11.58	78.22±20.97	64.64±21.90	96.89±20.21	53.17±13.30	67.27±15.37	61.37±18.03
**K/Na ratio**	**4.74**	**2.16**	**3.85**	**4.75**	**5.80**	**7.03**	**5.25**	**5.86**	**2.08**

The percentage of live cells in each cell passage as determined by Trypan Blue dye exclusion test and the combined LIVE/DEAD™ Assay are shown in the first two rows. In the following rows, the intracellular ionic concentrations of calcium, chlorine, potassium, magnesium, sodium, phosphorous, sulfur and K/Na ratio are shown. In all cases, both the mean values and standard deviation are shown for each cell passage and technique. Ionic concentrations are expressed in millimoles of each element per kilogram of cell dry weight.

**Table 2:** Statistical *p* values for the comparisons of the cell viability levels as determined by trypan blue dye exclusion test, and LIVE/DEAD™ Cell viability assay and the intracellular ionic concentration of calcium, chlorine, potassium, magnesium, sodium, phosphorous and sulfur in 9 consecutive cell passages of TMJF.

	P1–P2	P2–P3	P3–P4	P4–P5	P5–P6	P6–P7	P7–P8	P8–P9	All (Kendall W)
TRYPÁN BLUEExclusion test	0.1128	0.0264	0.9158	0.0264	0.0264	0.0264	0.1128	0.0264	0.0001*
**LIVE/DEAD™ Cell Viability Assay**	0.7532	0.1730	0.9165	0.2489	0.7150	1.0000	0.0747	0.1730	0.3594
**Ca**	0.9932	0.0401	0.9501	0.0028*	0.9262	0.7221	0.8733	0.0180	0.0001*
**Cl**	0.3926	0.0000*	0.0312	0.2162	0.0005*	0.0001*	0.0051*	0.6822	0.0000*
**K**	0.0001*	0.3339	0.0002*	0.1635	0.0406	0.0000*	0.0000*	0.0000*	0.0000*
**Mg**	0.1877	0.4220	0.0003*	0.0106	0.3130	0.0001*	0.0000*	0.0026*	0.0000*
**Na**	0.0004*	0.0000*	0.0114	0.0092*	0.8508	0.0360	0.0000*	0.0018*	0.0000*
**P**	0.0013*	0.1157	0.0003*	0.0000*	0.0383	0.0000*	0.0000*	0.0007*	0.0000*
**S**	0.0001*	0.0294	0.0007*	0.0029*	0.0000*	0.0000*	0.0004*	0.0740	0.0000*

Pairwise comparisons between two consecutive cell passages were performed by using the Wilcoxon non-parametric test. The global significant differences among the 9 cell passages were analyzed by the W test of Kendall. Statistically significant *p* values are shown with asterisks (*).

In the second place, the results obtained with Live/Dead™ Viability/Cytotoxicity assays showed that the lowest rate of viability corresponded to P1 and P2 (Table 1). From there, an increasing cell viability trend was found from P3 to P7, with a final decrease until P9. No statistical differences were found for any of the statistical comparisons carried out (Table 2).

**Figure 1:**
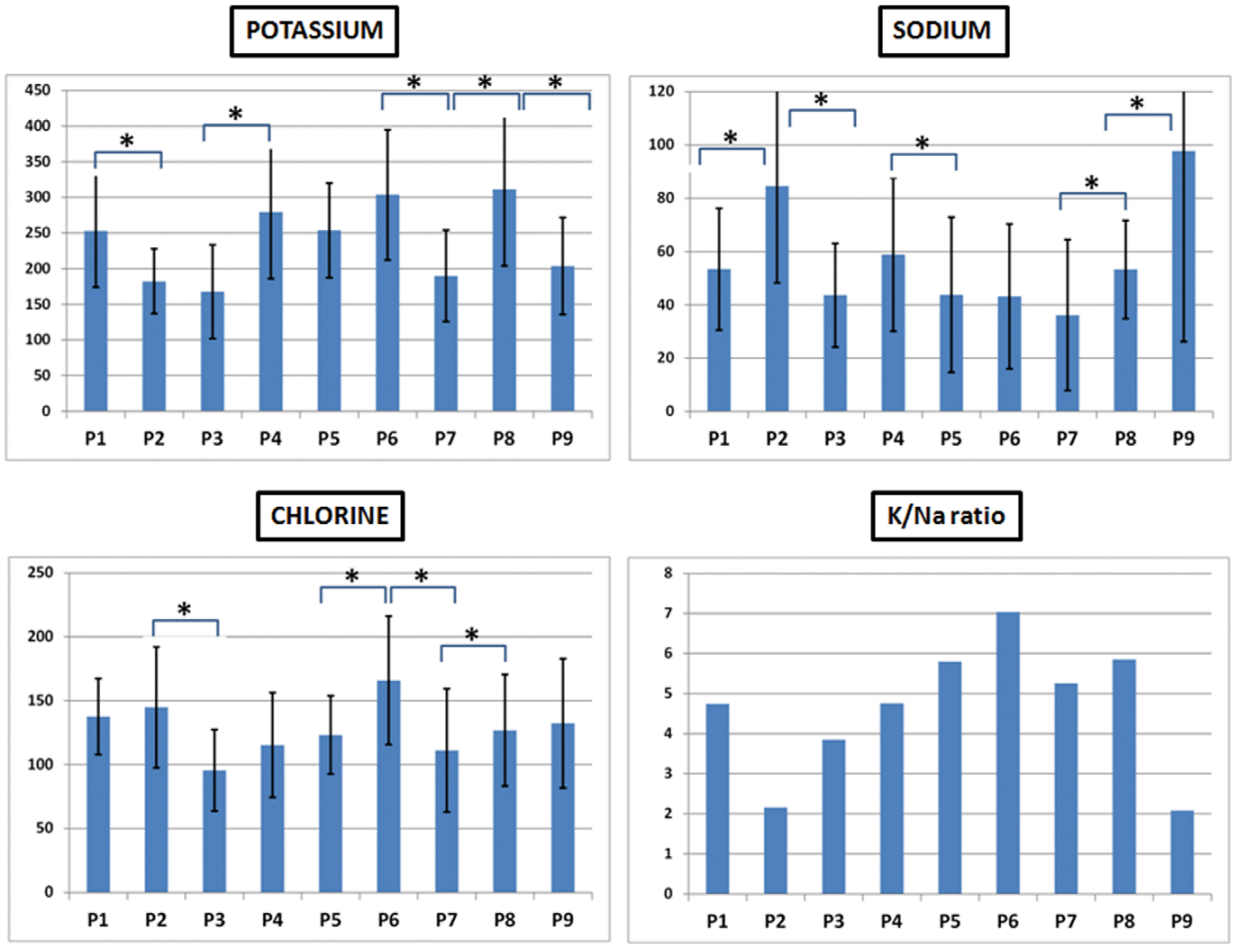
Intracellular ionic concentrations of potassium, sodium, chlorine and K/Na ratio of 9 consecutive cell passages of TMJF cells. Statistically significant differences between two consecutive cell passages are labeled with asterisks. All values are expressed as millimoles of each element per kilogram of cell dry weight and are shown as mean ± standard deviation.

In addition, the analysis of cell viability as determined by electron-probe X-ray microanalysis of 9 consecutive TMJF subcultures showed that the K/Na ratio was high in all cell passages, ranging from 2.08 at P9 to 7.02 at P6, showing increasing levels from P2 to P6 and a trend to decrease from P6 to P9 (Table 1 and Figure 1). When specific intracellular elements were analyzed (Ca, Cl, K, Mg, Na, P and S), we found statistically significant differences among all 9 cells passages globally analyzed (p = 0.000 for the W test of Kendall), suggesting that the intracellular concentration of these elements significantly varied among all 9 cell passages (Table 2). The analysis of intracellular K concentrations showed some significant variations, being especially remarkable a decrease found from P1 to P2, an increase from P3 to P4 and a final decrease from P8 to P9 (Figure 1). The intracellular Na concentrations showed a significant increase at P2 and at the final passages P8 and P9. When chlorine concentrations were microanalyzed, we found that the highest ionic levels were found at P6, with a significant increase from P5 to P6 and a significant decrease from P6 to P7 (Figure 1). Interestingly, the concentration of phosphorous significantly varied at some cell passages. Among others, a significant decrease was detected from P1 to P2 and from P8 to P9. Similarly, the concentration of Mg significantly decreased at P9. Regarding the concentrations of S, this element reached the highest concentrations at P6, with a significant increase from P5 to P6 and a significant decrease from P6 to P7. The concentrations of calcium significantly decreased from P4 to P5.

**Figure 2:**
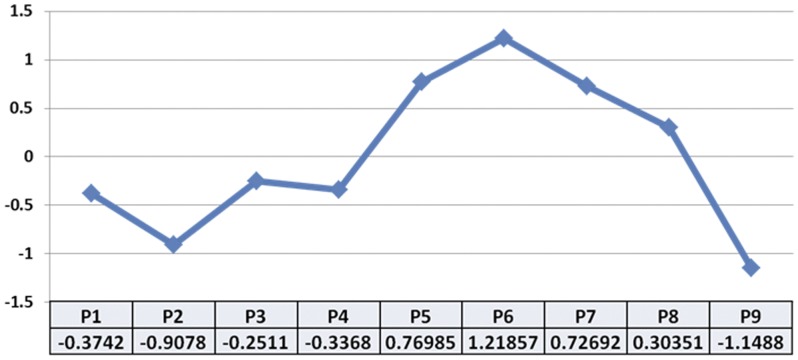
Cell viability index of 9 consecutive cell passages (P1 to P9) of TMJF obtained by normalization of trypan blue dye test, LIVE/DEAD™ Cell viability assay and EPXMA (K/Na ratio) values.

Based on the results of trypan blue exclusion tests, Live/Dead™ Viability/Cytotoxicity Assay and the intracellular K/Na concentrations as determined by electron-probe X-ray microanalysis assays, we calculated the cell viability index for each cell passage. As shown in Figure 2, the highest cell viability index corresponded to the sixth cell passage (P6), followed by P5 and P7, with the lowest values corresponding to P9, followed by P2.

**Figure 3:**
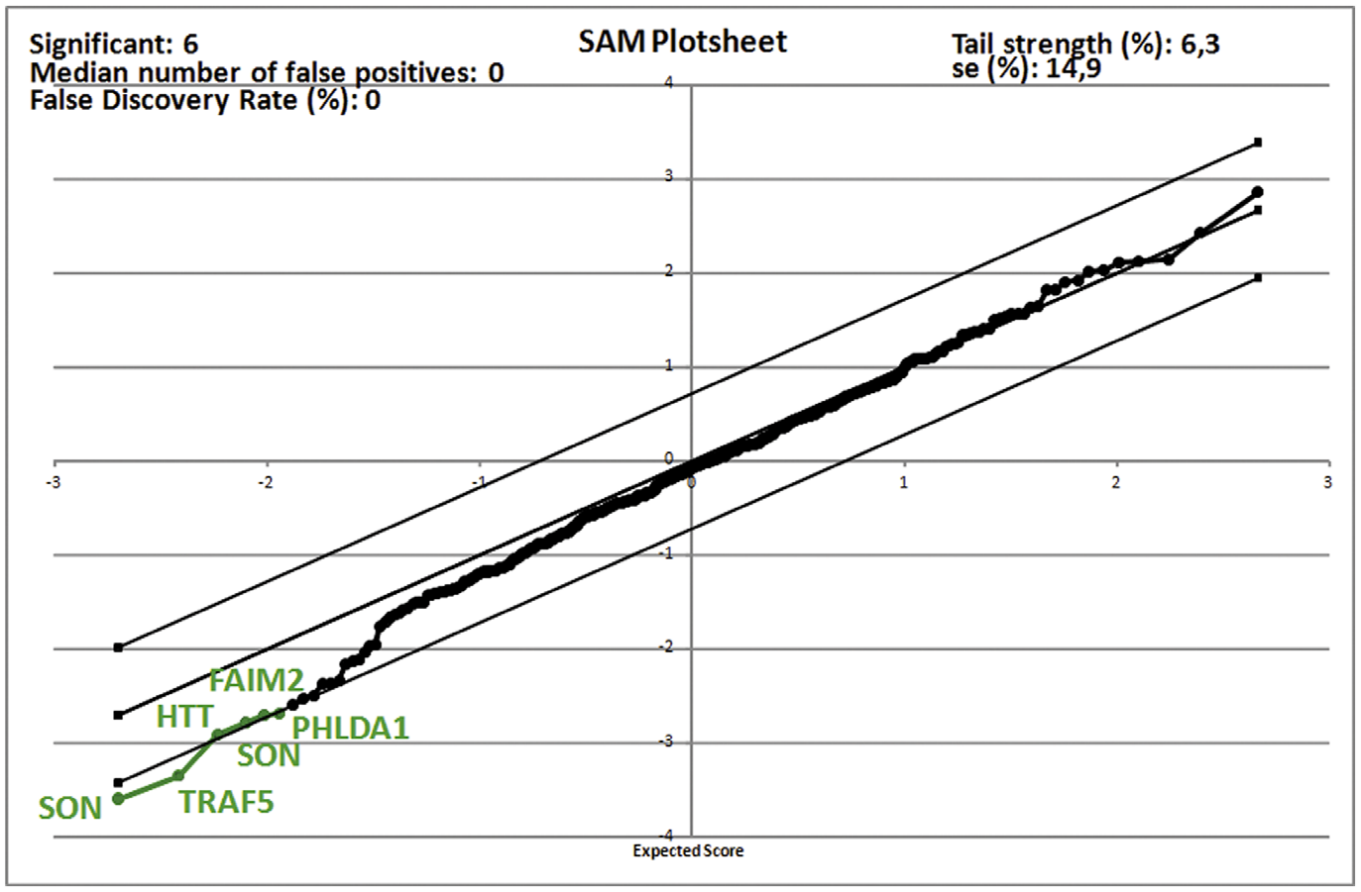
Analysis of genes significantly associated to the cell viability index as determined by the Significance Analysis of Microarray (SAM) software. All significant genes are represented in green color on the plot.

Finally, the SAM analysis of microarray-determined gene expression values (Figure 3) demonstrated that 6 probe sets corresponding to 5 genes were inversely associated to the cell viability index, including 2 genes with a role in apoptosis induction (TRAF5, GO:0006915 apoptotic process; and PHLDA1, GO:0006917 induction of apoptosis and GO:0006915 apoptotic process) and 3 genes with a role in apoptosis inhibition (SON, HTT and FAIM2, GO:0006916 anti-apoptosis for the 3 genes). The highest expression of all these 5 genes significantly correlated with the lowest cell viability index. None of the genes in the array was positively correlated with the cell viability index.

### Expression of ECM Components Along 9 Consecutive Cell Passages of TMJF

The analysis of 49 fibrillar components of the fibrocartilage ECM showed a significant diminution of the expression of several genes along the nine consecutive cell passages, including two genes encoding for collagen type I and procollagen (COL1A1, COL1A2, PLOD1 and PLOD2) representing 32% of all fibrillar genes. The rest of the fibrillar components genes (68%) did not show any relevant significance (Table 3).

Once we analyzed the fibrillar components of the fibrocartilage ECM, we performed the analysis of non-fibrillar components of the ECM by quantifying the expression of the main glucosaminoglycans (GAGs), proteoglycans (PGs), multiadhesive gluproteins (MGPs) and other ECM genes (Table 3). As the result of this, we found that 6 GAGs genes (17% of genes) notably decreased after continuous cell passaging, including some genes related to chondroitin-sulfate, hyaluronic acid and heparan-sulfate synthesis such as CHST12, CSGALNACT1, HAS1 and HS6ST2. However, the gene expression of two genes (5% of all genes) with a role in heparan-sulfate metabolism were increased (correlation coefficient r>0.7) and the rest of the genes (78% of all genes) did not differ. The study of 13 markers related to PGs synthesis demonstrated that the expression of 3 genes, aggrecan, biglycan and decorin (24% of all genes) significantly decreased after nine consecutive subcultures, although 10 genes (76% of all genes) did not show any variation. Regarding the study of 19 MGPs genes, 3 of them (16%) showed a decreasing tendency, including nidogen, osteonectin and tenascin (NID2, SPARC and TNC), whilst two genes (10%) encoding for laminin (LAMA4 and LAMB1) showed the inverse tendency along cell passaging. Finally, the analysis of other genes with a role in cartilage generation and metabolism showed a significant decrease of 6 genes (43% of all genes) including CHODL, CILP, COMP, CRTA1 and FMOD.

**Table 3:** Expression of ECM components along 9 consecutive cell passages of TMJF.

ECM COMPONENT	GENE SYMBOL	GENE TITLE	P1	P2	P3	P4	P5	P6	P7	P8	P8	CELL PASSAGE CORRELATION
**ECM-F**	**COL1A1**	**collagen, type I, alpha 1**	3279.8	3075.5	3293.1	2358.0	2736.6	2578.3	2679.0	2516.4	2613.2	−0.71748^**^
**ECM-F**	**COL1A2**	**collagen, type I, alpha 2**	5854.4	5996.2	5728.2	4395.6	5150.3	4775.4	4567.0	4413.0	4738.0	−0.79913^**^
**ECM-F**	**COL2A1**	**collagen, type II, alpha 1**	4.0	3.5	5.3	2.2	4.6	2.7	5.4	4.4	2.5	−**0.09767**
**ECM-F**	**COL3A1**	**collagen, type III, alpha 1**	5159.0	4705.3	4466.0	3314.3	4243.6	3512.9	3699.2	2457.6	3690.6	−0.78038^**^
**ECM-F**	**COL4A1**	**collagen, type IV, alpha 1**	1179.6	367.3	422.3	247.3	381.8	192.6	248.9	104.1	251.5	−0.70610^**^
**ECM-F**	**COL4A2**	**collagen, type IV, alpha 2**	1056.0	409.6	426.8	255.4	404.0	222.9	263.4	169.5	229.6	−0.74250^**^
**ECM-F**	**COL4A3**	**collagen, type IV, alpha 3 (Goodpasture antigen)**	6.0	4.9	7.1	4.2	11.6	6.7	10.3	2.8	4.6	−**0.04759**
**ECM-F**	**COL4A3BP**	**collagen, type IV, alpha 3 (Goodpasture antigen) binding protein**	226.8	231.2	216.9	229.7	237.3	211.0	229.3	308.3	284.4	**0.65648**
**ECM-F**	**COL4A4**	**collagen, type IV, alpha 4**	28.9	24.1	24.3	16.2	34.3	22.5	37.7	4.7	7.8	−**0.44900**
**ECM-F**	**COL4A5**	**collagen, type IV, alpha 5**	21.0	25.6	29.2	38.4	56.3	36.1	35.4	3.1	59.2	**0.25229**
**ECM-F**	**COL4A6**	**collagen, type IV, alpha 6**	3.5	3.4	6.3	4.8	2.6	3.9	3.7	3.7	6.0	**0.18275**
**ECM-F**	**COL5A1**	**collagen, type V, alpha 1**	962.5	761.2	615.0	496.3	603.7	559.3	486.1	411.1	285.0	−0.91331^**^
**ECM-F**	**COL5A2**	**collagen, type V, alpha 2**	2091.1	1751.4	1810.1	1530.2	1880.2	1634.0	1768.3	1113.4	1499.1	−0.70044^**^
**ECM-F**	**COL5A3**	**collagen, type V, alpha 3**	260.4	100.1	82.0	42.7	91.3	52.6	75.9	13.8	38.4	−0.73098^**^
**ECM-F**	**COL6A1**	**collagen, type VI, alpha 1**	1106.8	1389.4	1248.4	1014.0	1156.2	1208.1	1085.4	1026.2	1235.6	−**0.26779**
**ECM-F**	**COL6A2**	**collagen, type VI, alpha 2**	2166.0	2565.0	2214.8	1873.5	2009.1	1830.4	1778.9	2020.1	1809.2	−0.71504^**^
**ECM-F**	**COL6A3**	**collagen, type VI, alpha 3**	6106.0	7130.0	6994.3	5423.8	5716.3	5367.2	4923.1	5855.6	5732.4	−**0.59391**
**ECM-F**	**COL6A6**	**collagen type VI alpha 6**	43.7	14.2	5.5	7.8	11.0	7.0	3.4	13.6	5.1	−**0.59583**
**ECM-F**	**COL7A1**	**collagen, type VII, alpha 1**	32.1	18.4	28.8	20.6	22.0	20.7	22.4	14.4	24.2	−**0.48864**
**ECM-F**	**COL8A1**	**collagen, type VIII, alpha 1**	147.8	173.6	137.8	179.0	335.1	255.9	395.3	73.6	98.8	**0.04052**
**ECM-F**	**COL8A2**	**collagen, type VIII, alpha 2**	631.7	713.7	860.3	587.2	678.8	461.6	742.9	124.1	869.6	−**0.23496**
**ECM-F**	**COL9A1**	**collagen, type IX, alpha 1**	0.6	0.9	1.7	1.6	1.5	2.9	1.3	1.1	0.6	**0.08364**
**ECM-F**	**COL9A2**	**collagen, type IX, alpha 2**	9.9	11.4	13.2	10.8	12.3	8.6	9.0	11.6	14.7	**0.21250**
**ECM-F**	**COL9A3**	**collagen, type IX, alpha 3**	1.1	2.6	3.7	2.8	5.0	1.7	1.6	0.6	1.6	−**0.29908**
**ECM-F**	**COL10A1**	**collagen, type X, alpha 1**	6.6	7.0	11.7	18.7	30.1	29.2	19.8	18.7	4.1	**0.24673**
**ECM-F**	**COL11A1**	**collagen, type XI, alpha 1**	1349.1	818.1	926.0	1130.2	789.7	354.4	374.7	130.1	463.1	−0.85245^**^
**ECM-F**	**COL11A2**	**collagen, type XI, alpha 2**	18.0	13.2	26.3	22.9	20.8	15.0	17.2	18.1	22.0	**0.05105**
**ECM-F**	**COL12A1**	**collagen, type XII, alpha 1**	1640.2	2159.7	2231.6	1505.3	1420.5	1133.5	1374.1	755.6	1360.6	−0.73029^**^
**ECM-F**	**COL13A1**	**collagen, type XIII, alpha 1**	16.0	11.4	23.2	17.9	10.9	27.4	28.1	71.3	19.6	**0.52991**
**ECM-F**	**COL14A1**	**collagen, type XIV, alpha 1**	493.2	460.0	446.5	340.1	288.0	354.5	300.7	330.2	822.4	**0.17915**
**ECM-F**	**COL15A1**	**collagen, type XV, alpha 1**	919.5	1288.9	1699.7	832.7	691.2	130.9	164.5	116.6	215.5	−0.81040^**^
**ECM-F**	**COL16A1**	**collagen, type XVI, alpha 1**	331.5	315.2	261.4	311.7	249.4	227.1	175.9	135.2	208.2	−0.88129^**^
**ECM-F**	**COL17A1**	**collagen, type XVII, alpha 1**	2.0	1.0	2.6	1.7	1.6	0.9	1.4	1.4	2.2	−**0.06571**
**ECM-F**	**COL18A1**	**collagen, type XVIII, alpha 1**	59.0	52.0	50.9	46.3	53.9	34.8	41.0	28.9	45.7	−0.73427^**^
**ECM-F**	**COL19A1**	**collagen, type XIX, alpha 1**	2.1	2.7	1.9	4.9	3.2	3.8	3.2	7.2	3.8	**0.62029**
**ECM-F**	**COL20A1**	**collagen, type XX, alpha 1**	4.7	4.1	6.1	8.3	4.5	6.8	8.6	1.4	3.5	−**0.18773**
**ECM-F**	**COL21A1**	**collagen, type XXI, alpha 1**	85.6	96.4	84.8	258.9	218.7	117.3	125.6	76.9	107.9	−**0.02054**
**ECM-F**	**COL22A1**	**collagen, type XXII, alpha 1///similar to collagen, type XXII, alpha 1**	3.3	3.6	4.0	5.1	3.0	2.1	4.9	4.4	3.9	**0.17585**
**ECM-F**	**COL23A1**	**collagen, type XXIII, alpha 1**	10.3	7.0	2.9	3.2	5.9	8.6	1.6	9.1	6.9	−**0.06547**
**ECM-F**	**COL24A1**	**collagen, type XXIV, alpha 1**	13.7	10.5	7.3	10.7	11.9	9.5	9.1	2.3	10.3	−**0.50646**
**ECM-F**	**COL25A1**	**collagen, type XXV, alpha 1**	1.6	3.5	3.5	4.8	4.4	2.4	2.9	0.3	3.5	−**0.18502**
**ECM-F**	**COL27A1**	**collagen, type XXVII, alpha 1**	45.0	43.5	36.4	33.4	51.3	25.7	39.1	12.9	102.8	**0.25054**
**ECM-F**	**COL28A1**	**collagen, type XXVIII, alpha 1**	3.8	3.1	4.2	6.2	3.2	4.6	5.3	5.2	0.9	−**0.14774**
**ECM-F**	**COL29A1**	**collagen, type XXIX, alpha 1**	3.7	2.5	3.5	3.9	5.5	3.0	4.3	0.7	4.7	−**0.02374**
**ECM-F**	**ELN**	**elastin**	882.1	324.8	269.8	76.4	143.7	263.7	202.2	301.4	88.0	−**0.60273**
**ECM-F**	**FBN1**	**fibrillin 1**	2900.0	2760.2	3164.1	2287.4	2471.8	2755.3	2283.6	3853.9	3268.2	**0.30977**
**ECM-F**	**FBN2**	**fibrillin 2**	20.7	10.1	9.3	10.4	18.3	8.3	11.9	7.2	8.2	−**0.53492**
**ECM-F**	**PLOD1**	**procollagen-lysine, 2-oxoglutarate 5-dioxygenase 3**	525.3	675.4	525.7	520.7	537.9	543.2	476.4	502.0	350.0	−0.70698^**^
**ECM-F**	**PLOD2**	**procollagen-lysine, 2-oxoglutarate 5-dioxygenase 2**	913.9	1041.6	929.6	644.7	945.2	802.8	892.1	566.0	483.9	−0.72645^**^
**ECM-GAGS**	**CHPF**	**chondroitin polymerizing factor**	125.6	135.0	143.5	139.1	149.6	164.7	165.7	232.5	147.2	**0.65018**
**ECM-GAGS**	**CHST1**	**carbohydrate (keratan sulfate Gal-6) sulfotransferase 1**	18.6	19.3	20.2	26.2	17.3	10.6	13.4	19.1	9.6	−**0.57579**
**ECM-GAGS**	**CHST11**	**carbohydrate (chondroitin 4) sulfotransferase 11**	9.4	7.0	13.4	14.3	15.6	9.8	25.2	35.7	2.3	**0.34775**
**ECM-GAGS**	**CHST12**	**carbohydrate (chondroitin 4) sulfotransferase 12**	237.5	223.5	213.8	250.9	240.5	185.8	187.5	192.6	184.0	−0.73573^**^
**ECM-GAGS**	**CHST13**	**carbohydrate (chondroitin 4) sulfotransferase 13**	3.3	4.4	9.0	10.1	4.8	4.6	5.6	5.7	1.0	−**0.29187**
**ECM-GAGS**	**CHST3**	**carbohydrate (chondroitin 6) sulfotransferase 3**	88.7	82.7	85.2	92.1	94.0	100.7	98.5	90.4	73.1	−**0.02084**
**ECM-GAGS**	**CHSY1**	**chondroitin sulfate synthase 1**	703.7	608.2	576.9	602.8	633.0	613.5	656.3	670.8	482.8	−**0.37902**
**ECM-GAGS**	**CHSY3**	**chondroitin sulfate synthase 3**	173.6	110.9	102.8	118.1	152.4	97.3	120.1	70.0	90.1	−**0.63964**
**ECM-GAGS**	**CSGALNACT1**	**chondroitin sulfate N-acetylgalactosaminyltransferase 1**	1680.9	1378.0	1343.5	611.5	1014.7	802.1	752.5	712.7	492.4	−0.87273^**^
**ECM-GAGS**	**CSGALNACT2**	**chondroitin sulfate N-acetylgalactosaminyltransferase 2**	439.4	491.7	468.8	397.8	430.4	396.3	516.0	403.5	431.9	−**0.21687**
**ECM-GAGS**	**CSGLCA-T**	**chondroitin sulfate glucuronyltransferase**	165.5	190.4	167.9	201.6	219.4	202.6	181.1	246.5	184.4	**0.48223**
**ECM-GAGS**	**CSECM-PGS4**	**chondroitin sulfate proteoglycan 4**	41.2	77.8	105.5	97.5	76.6	78.9	60.7	72.9	69.3	−**0.02538**
**ECM-GAGS**	**CSECM-PGS4LYP1///CSECM-PGS4LYP2**	**chondroitin sulfate proteoglycan 4-like, Y-linked pseudogene 1///chondroitin sulfate proteoglycan 4-like, Y-linked pseudogene 2**	1.6	5.7	2.4	1.2	2.9	1.1	1.3	1.0	1.2	−**0.53663**
**ECM-GAGS**	**CSECM-PGS5**	**chondroitin sulfate proteoglycan 5 (neuroglycan C)**	3.7	5.6	5.5	5.5	4.0	5.1	5.0	1.1	4.4	−**0.38941**
**ECM-GAGS**	**DSE**	**dermatan sulfate epimerase**	217.7	176.1	185.9	270.4	252.7	266.3	256.0	199.9	245.0	**0.40368**
**ECM-GAGS**	**DSEL**	**dermatan sulfate epimerase-like**	161.5	140.1	150.3	192.9	122.4	115.8	136.9	136.2	157.5	−**0.25984**
**ECM-GAGS**	**HAS1**	**hyaluronan synthase 1**	92.9	47.4	70.1	3.8	10.7	14.1	4.3	22.1	3.0	−0.77348^**^
**ECM-GAGS**	**HAS2**	**hyaluronan synthase 2**	205.5	196.1	245.3	248.5	110.0	324.5	92.4	44.9	77.9	−**0.57677**
**ECM-GAGS**	**HAS3**	**hyaluronan synthase 3**	15.1	16.6	14.9	12.0	17.3	13.8	16.0	22.0	35.2	**0.65743**
**ECM-GAGS**	**HGSNAT**	**heparan-alpha-glucosaminide N-acetyltransferase**	143.2	160.0	148.1	132.8	140.5	146.7	148.6	163.3	162.0	**0.43871**
**ECM-GAGS**	**HS2ST1**	**heparan sulfate 2-O-sulfotransferase 1**	135.6	128.8	153.6	211.6	168.3	175.3	216.9	241.0	185.1	0.75056^*^
**ECM-GAGS**	**HS3ST1**	**Heparan sulfate 3-O-sulfotransferase-1 precursor (3OST1)**	7.8	5.5	7.6	9.6	8.9	4.8	3.3	1.5	4.8	−**0.63282**
**ECM-GAGS**	**HS3ST2**	**heparan sulfate (glucosamine) 3-O-sulfotransferase 2**	33.6	33.9	20.8	30.7	26.1	16.0	17.9	12.4	26.1	−**0.67088**
**ECM-GAGS**	**HS3ST3A1**	**heparan sulfate (glucosamine) 3-O-sulfotransferase 3A1**	31.7	26.8	27.8	33.5	28.2	34.4	41.0	38.0	52.4	0.80926^*^
**ECM-GAGS**	**HS3ST3B1**	**heparan sulfate (glucosamine) 3-O-sulfotransferase 3B1**	128.2	98.3	112.8	125.0	93.1	73.9	95.0	65.1	89.7	−0.73007^**^
**ECM-GAGS**	**HS3ST4**	**heparan sulfate (glucosamine) 3-O-sulfotransferase 4**	10.9	8.1	3.3	13.6	12.6	7.9	2.0	7.0	6.2	−**0.35097**
**ECM-GAGS**	**HS3ST5**	**heparan sulfate (glucosamine) 3-O-sulfotransferase 5**	11.6	8.6	14.0	13.6	12.6	14.8	10.4	18.7	10.1	**0.27891**
**ECM-GAGS**	**HS3ST6**	**heparan sulfate (glucosamine) 3-O-sulfotransferase 6**	3.4	0.9	6.1	3.4	1.2	4.3	6.6	0.9	5.7	**0.22829**
**ECM-GAGS**	**HS6ST1**	**heparan sulfate 6-O-sulfotransferase 1**	97.9	73.6	78.3	57.5	85.3	56.7	80.1	48.3	40.7	−0.73140^**^
**ECM-GAGS**	**HS6ST2**	**heparan sulfate 6-O-sulfotransferase 2**	9.7	8.9	3.6	5.9	4.4	0.9	5.0	3.0	2.6	−0.76246^**^
**ECM-GAGS**	**HS6ST3**	**heparan sulfate 6-O-sulfotransferase 3///similar to heparan sulfate 6-O-sulfotransferase 3**	4.4	2.8	3.3	4.3	7.7	5.1	5.0	1.2	2.0	−**0.23524**
**ECM-GAGS**	**NDST1**	**N-deacetylase/N-sulfotransferase (heparan glucosaminyl) 1**	76.1	74.1	80.4	84.1	81.4	74.6	75.7	90.0	69.3	**0.01531**
**ECM-GAGS**	**NDST2**	**N-deacetylase/N-sulfotransferase (heparan glucosaminyl) 2**	76.7	58.3	58.0	77.7	66.7	58.0	55.4	63.4	82.7	**0.06350**
**ECM-GAGS**	**NDST3**	**N-deacetylase/N-sulfotransferase (heparan glucosaminyl) 3**	4.9	2.1	4.7	6.4	5.6	6.1	1.5	1.1	6.4	−**0.06904**
**ECM-GAGS**	**NDST4**	**N-deacetylase/N-sulfotransferase (heparan glucosaminyl) 4**	3.0	2.6	2.8	2.2	4.3	1.6	0.8	0.4	3.6	−**0.31032**
**ECM-MGPS**	**CHI3L1**	**chitinase 3-like 1 (cartilage glycoprotein-39)**	5426.4	6292.7	5125.1	4433.1	4875.7	4486.7	4597.7	4416.5	4587.1	−0.73492^**^
**ECM-MGPS**	**FN1**	**fibronectin 1**	3619.5	4280.1	4404.0	3245.8	3650.1	3647.1	3335.1	3947.2	3699.1	−**0.28325**
**ECM-MGPS**	**LAMA1**	**laminin, alpha 1**	54.2	78.6	59.3	59.6	62.9	43.7	52.0	106.3	80.6	**0.37824**
**ECM-MGPS**	**LAMA2**	**laminin, alpha 2**	128.4	175.5	173.1	187.4	135.4	111.3	107.4	165.1	77.6	−**0.53955**
**ECM-MGPS**	**LAMA3**	**laminin, alpha 3**	6.3	10.5	12.0	13.3	11.6	16.1	14.5	30.7	10.0	**0.55274**
**ECM-MGPS**	**LAMA4**	**laminin, alpha 4**	213.5	213.9	186.9	204.3	248.5	271.6	241.5	249.5	323.2	0.80093^*^
**ECM-MGPS**	**LAMA5**	**KIAA0533 protein**	26.4	43.2	54.8	45.2	37.9	33.3	35.5	36.1	48.0	**0.07749**
**ECM-MGPS**	**LAMB1**	**laminin, beta 1**	966.6	1027.8	1089.8	1201.4	1234.5	938.6	1187.8	1262.1	1607.2	0.72252^*^
**ECM-MGPS**	**LAMB2**	**laminin, beta 2 (laminin S)**	424.3	418.9	470.6	341.8	470.2	340.7	345.0	372.4	343.5	−**0.59391**
**ECM-MGPS**	**LAMB2L**	**laminin, beta 2-like**	12.9	12.2	13.4	16.4	9.5	6.6	14.5	10.6	18.9	**0.14682**
**ECM-MGPS**	**LAMB3**	**laminin, beta 3**	25.5	20.5	20.1	26.8	39.0	24.9	28.3	30.5	23.6	**0.29335**
**ECM-MGPS**	**LAMB4**	**laminin, beta 4**	6.8	3.5	9.1	7.1	5.1	5.1	5.8	5.8	5.8	−**0.17541**
**ECM-MGPS**	**LAMC1**	**laminin, gamma 1 (formerly LAMB2)**	1531.5	1339.8	1688.1	1552.6	1646.6	1625.8	1619.1	2620.0	1566.9	**0.49174**
**ECM-MGPS**	**LAMC2**	**laminin, gamma 2**	11.4	15.9	14.6	10.4	11.8	13.2	8.2	13.3	13.1	−**0.21771**
**ECM-MGPS**	**LAMC3**	**laminin, gamma 3**	9.7	6.5	10.1	7.9	7.0	8.9	11.0	8.4	12.3	**0.46188**
**ECM-MGPS**	**NID1**	**nidogen 1_ENTACTIN**	757.2	684.8	762.9	637.4	651.6	612.7	672.2	318.0	859.9	−**0.27221**
**ECM-MGPS**	**NID2**	**nidogen 2 (osteonidogen)**	1749.3	1520.4	1241.2	1608.2	1690.2	919.2	1175.6	574.4	1076.8	−0.73727^**^
**ECM-MGPS**	**SPARC**	**secreted protein, acidic, cysteine-rich (osteonectin)**	4099.1	3863.9	4233.4	3108.5	3785.4	3612.2	3730.2	2866.4	2952.8	−0.74368^**^
**ECM-MGPS**	**TNC**	**Tenascin**	1761.7	2471.1	2131.0	1738.7	1515.9	1604.2	1662.7	1183.1	1264.1	−0.78805^**^
**ECM-OG**	**BMP4**	**bone morphogenetic protein 4**	22.5	13.2	27.1	4.3	29.9	22.1	48.2	21.8	13.3	**0.18063**
**ECM-OG**	**CHAD**	**chondroadherin**	1.0	0.7	2.7	2.9	2.1	1.0	2.5	1.5	6.7	**0.57996**
**ECM-OG**	**CHADL**	**chondroadherin-like**	6.9	15.4	23.6	13.3	25.0	13.2	15.8	17.8	19.9	**0.35620**
**ECM-OG**	**CHODL**	**chondrolectin**	55.6	43.4	35.7	37.8	3.2	7.3	8.1	21.0	8.4	−0.81633^**^
**ECM-OG**	**CILP**	**cartilage intermediate layer protein, nucleotide pyrophosphohydrolase**	157.0	99.3	56.4	47.9	29.9	34.3	31.7	21.9	59.9	−0.72203^**^
**ECM-OG**	**CILP2**	**cartilage intermediate layer protein 2**	18.5	21.6	24.0	20.3	12.1	14.5	17.3	4.9	10.0	−0.77237^**^
**ECM-OG**	**COMP**	**cartilage oligomeric matrix protein**	927.6	705.0	598.0	257.9	765.7	321.5	591.0	178.2	70.9	−0.77008^**^
**ECM-OG**	**CRTAC1**	**cartilage acidic protein 1**	15.4	9.5	9.9	8.6	8.3	6.2	8.8	2.5	4.6	−0.85984^**^
**ECM-OG**	**CRTAP**	**cartilage associated protein**	975.9	909.2	906.1	984.7	1037.5	987.6	799.9	850.9	1003.7	−**0.15987**
**ECM-OG**	**FMOD**	**fibromodulin**	3560.6	3074.9	2821.7	1436.2	2224.1	1656.3	2721.3	760.5	1127.3	−0.79277^**^
**ECM-OG**	**MATN1**	**matrilin 1, cartilage matrix protein**	8.2	8.8	7.5	4.6	6.5	3.5	11.5	2.8	7.1	−**0.26005**
**ECM-OG**	**MATN2**	**matrilin 2**	316.7	284.7	455.0	506.2	182.1	177.9	140.9	218.6	339.5	−**0.38274**
**ECM-OG**	**MATN3**	**matrilin 3**	15.0	10.9	11.0	19.1	16.9	15.3	11.3	7.1	8.8	−**0.45727**
**ECM-OG**	**MATN4**	**matrilin 4**	20.6	14.9	22.4	11.7	14.3	11.4	12.5	8.1	18.5	−**0.47581**
**ECM-PGS**	**ACAN**	**aggrecan**	94.7	178.3	108.7	100.1	96.0	45.0	74.8	63.2	50.8	−0.73427^**^
**ECM-PGS**	**BGN**	**biglycan**	799.9	913.9	702.3	641.9	660.0	372.5	523.1	107.6	498.4	−0.80843^**^
**ECM-PGS**	**DCN**	**decorin**	6410.6	6324.6	6405.2	4944.5	5538.7	5202.4	5137.9	4938.7	5362.0	−0.77592^**^
**ECM-PGS**	**HSECM-PGS2**	**heparan sulfate proteoglycan 2_PERLECAN**	307.2	292.8	255.1	248.7	240.7	257.2	237.3	429.5	221.6	**0.02906**
**ECM-PGS**	**LUM**	**lumican**	2483.0	3093.5	3560.5	2547.2	2188.4	2405.9	2347.6	1957.7	2443.7	−**0.57811**
**ECM-PGS**	**NCAN**	**neurocan**	8.7	4.6	7.0	2.5	3.5	4.2	2.5	6.6	8.1	−**0.06403**
**ECM-PGS**	**SDC1**	**syndecan 1**	78.8	69.0	98.6	142.3	38.3	89.3	67.3	83.6	198.3	**0.39083**
**ECM-PGS**	**SDC2**	**syndecan 2**	858.6	563.4	509.6	562.8	626.6	601.5	629.4	601.2	715.0	−**0.08092**
**ECM-PGS**	**SDC3**	**syndecan 3**	43.1	51.4	82.1	62.2	37.9	36.8	38.4	45.2	51.5	−**0.30505**
**ECM-PGS**	**SDC4**	**syndecan 4**	1041.6	1140.8	1008.7	1028.9	878.0	861.6	889.8	777.5	1257.2	−**0.19091**
**ECM-PGS**	**SDCBP**	**syndecan binding protein (syntenin)**	4272.8	3965.3	4684.0	3753.7	3960.8	3546.2	3621.9	4098.4	5156.0	**0.14011**
**ECM-PGS**	**SDCBP2**	**syndecan binding protein (syntenin) 2**	28.1	27.2	16.1	29.6	34.5	33.1	35.2	27.7	28.7	**0.36935**
**ECM-PGS**	**VCAN**	**versican**	2940.9	3089.2	3159.3	2274.0	1480.7	2212.0	1903.2	2406.6	2269.3	−**0.59644**

Each gene has been classified as **ECM-F** (ECM- fibrillar component), **ECM-GAGS** (ECM-glycosaminoglycans), **ECM-MGPS** (ECM- multiadhesive gluproteins), **ECM-OG** (other genes with a function in ECM), **ECM-PGS** (ECM-proteoglycans). The correlation between gene expression and cell passaging as determined by the Pearson (r) correlation test is shown in the last column. All genes with a positive correlation with cell passaging (r >0.700) are shown with asterisks (*)**.** Genes with a negative correlation with cell passaging (r <−0.700) are shown with double-asterisks (**).

### Analysis of Cell Proliferation of 9 Consecutive Cell Passages of TMJF

**Figure 4:**
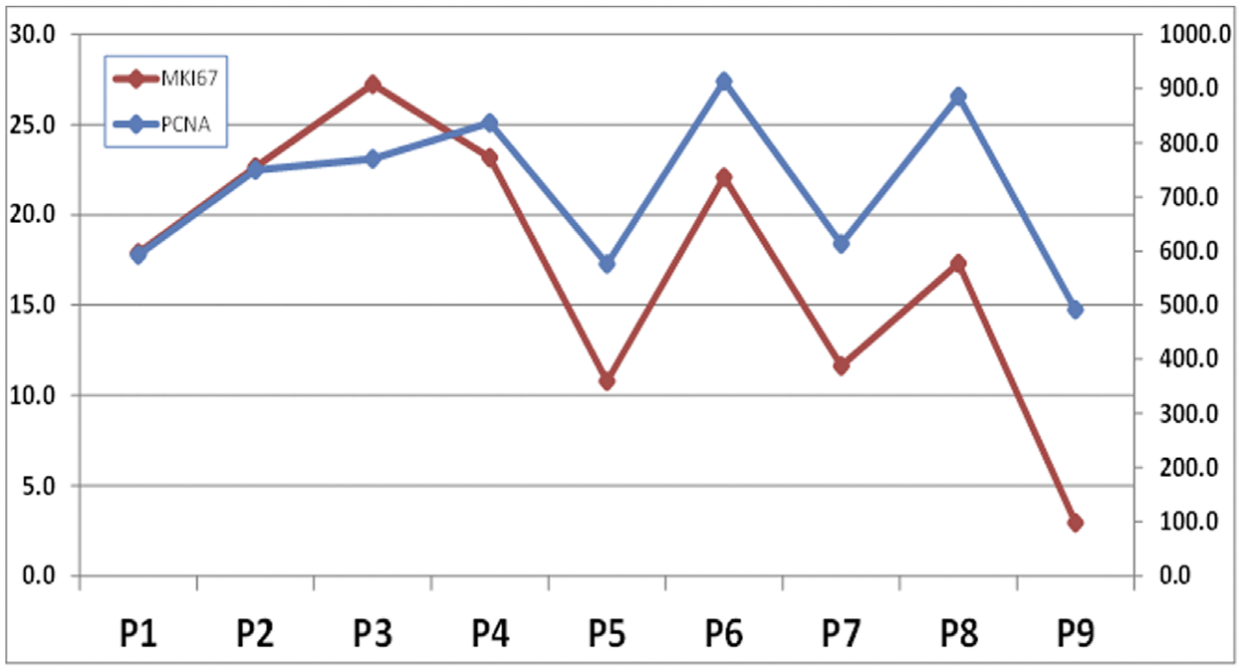
Analysis of cell proliferation of 9 TMJF cell passages by microarray. Average expression of the proliferation-related genes PCNA (in blue) and MKI67 (in red) are shown for each cell passage.

The mRNA expression of PCNA and MKI67 as determined by microarray analysis showed that the expression of both genes was high at most cell passages, with a reduction at P9 (Figure 4). The statistical analysis revealed that differences between passages were not significant, and a positive correlation was found between the expression levels of both PCNA and MKI67 (p = 0.0250 and r = 0.3907 for the correlation test).

## Discussion

A therapeutic advance in the treatment of TMJ pathological conditions could be the generation of biological substitutes of damaged discs generated by tissue engineering. Different models of engineered TMJ disc have been developed by using animal cells and different biomaterials and signaling [16]. In most of these studies, the key importance of an accurate cell viability determination has been established, since only viable cells should be used for TMJ disc tissue engineering [7], [9], [17]. Most current research works focused on TMJ disc regeneration have been developed by using animal cell sources including goat, calf, pig and rabbit [18]. Studies developed by Athanasiu [16] on goat costal chondrocytes demonstrated that the proliferative ability of the cells decreased after passage 5. In the present study, we have demonstrated that human TMJ cells were highly proliferative at most cell passages, with the lowest values found at P9. This could be possibly associated to a senescence process. It is important to highlight that most previous works have been performed in 5 or less cell passages.

In terms of cell viability, the classical idea that early passage cells are the most feasible for tissue engineering should be deeply considered, because it has been previously demonstrated that the earliest cell passages are frequently under adaptative conditions to ex vivo environments [10], [12] and they do not display the most accurate conditions to be used in tissue engineering protocols. In this sense, all cell sources should be previously characterized and deeply studied using a combination of highly sensitive techniques to select and determine the most accurate cell passage for regeneration protocols. In this milieu, we recently described an ACVL method to evaluate cell viability of human cells high accuracy [10]. In the present work, we used a combined approach based on the use of 3 different methods to determine a cell viability index at different levels: cell membrane integrity, cytoplasmic metabolism and intracellular ionic content.

Thus, our results revealed that the most viable cell passage of cultured TMJF could be P6, although P5 and P7 also showed good cell viability levels. Interestingly, the combination of classical protocols as trypan blue assays and precise techniques such as calcein/AM and ethidium homodimer-1 and electron-probe X-ray microanalysis, showed a good correlation between classical and novel assays to determine the cell behaviour of TMJF cells along nine subcultures. First, the trypan blue assay, a classical method that evaluates the cell membrane integrity evidenced high cell viability levels in all cell passages analyzed here, with the maximum cell viability at P6 which is in agreement with previous results obtained using WHJSC [10]. In the second place, we analyzed the enzymatic esterase cytoplasmatic activity and the membrane integrity by using calcein/AM and ethidium homodimer-1 assays. The results demonstrated that the most viable passage was P7 followed by P6 and P5. In contrast with trypan blue assays, this sensitive method showed that cell viability was very variable among the nine cell passages. In fact, P1 and P2 in junction with P9 were the less viable cell passages that were not efficiently detected by trypan blue assay. This may suggest that early stages of cell death in which the cell membrane has been not damaged could not be efficiently detected by trypan blue. For this reason, we hypothesize that the utilization of classical methods (trypan blue) should be always accompanied by more sensitive assays such as electron-probe X-ray microanalysis which allows us to determine qualitative and quantitative intracellular concentrations of key ions involved in cell viability. As we previously described, this method is highly sensitive for determination of the mechanisms of cell death that may occur after sequential cell passaging [12]. The link between intracellular levels of potassium, sodium, chlorine, calcium, sulfur, magnesium, phosphorous and cellular physiology has been deeply studied and it is clear that a close relationship exists between them. However, the intracellular ionic contents of TMJF and their relation with cell functions have not been previously described. In the first place, our results showed that the highest cell viability levels corresponded to P6 as determined by K/Na ratio followed by P8 and P5. Several studies [19], [20] previously confirmed the high accuracy of K/Na ratio as cell viability indicator with K, Na and Cl being the most sensitive and reliable marks. Strikingly, these results correlated very well with the classical and metabolic assays, thus confirming our results. Generally, low concentrations of intracellular potassium and high Na contents are associated to apoptosis, which can be detected in early pre-apoptotic stages by a decrease of Cl concentrations [10], [12]. In this regard, our results showed that an apoptotic process could be triggered from P1 to P2 and from P6 to P7, suggesting that P7 could not be viable. Moreover, high concentrations of sulfur and phosphorous and moderate concentrations of magnesium and calcium were also found at P6. From a microanalytical point of view, phosphorus is an element that is associated with cell mass and organic constituents, nucleic acids contents and the level of cellular phosphorylation, and cells characterized by a severe structural damage show a decrease in intracellular concentrations of phosphorus [21]. Therefore, the high values found at P6 would confirm the vital status of these cells, whereas the decrease at P7 could be related to a possible structural damage of these cells. In addition, magnesium has been associated with cellular ATP levels and a concentration decrease of magnesium is correlated with a decrease in cellular ATP concentration [22], phosphorylation, and DNA replication. The results obtained in this work suggest that cells of the TMJ articular disc of human adults would experience a decrease in ATP content in some cell passages, especially at P7. Similarly, the concentration of sulfur was high at P6. These results must be correlated with the participation of sulfur in the metabolism of sulfated proteins, proteoglycans and glycoproteins that are very important in these cells [23]. Finally, the lowest calcium values at P8 and the highest levels at P3 might be explained by cell viability alteration at these passages.

Once we determined cell viability at different levels, we obtained an average cell viability index that provides comprehensive information about the vital status of these cells. In summary, our combined cell viability analysis approach suggests that the most adequate cell passage for use in cell therapy could be P6, implying that these cells should preferably be used at this passage. The cell viability index results found that the most viable passages were P6 and P5. In these passages we found a perfect match with high levels of proliferation, low cell damage of the cell membrane as determined by trypan blue, and high metabolic activity along with an adequate equilibrium of sodium, magnesium, phosphorous, potassium, calcium, sulfur and chlorine levels. In contrast, P2 and P9 demonstrated to be the cell passages with compromised cell proliferation, ATP levels, cell volume, synthesis of sulfated proteins and evident alteration of the K/Na pump. This combined cell viability approach is likely to be much more accurate than the use of a single method or technique to evaluate cell viability, and gives valuable information on the metabolic and structural cell status. In fact, several studies previously carried out on articular cartilage found that the use of cultured human hyaline chondrocytes could not be clinically useful [7], [24], [25] Perhaps, some of these studies did not use the most viable cell passages and cell viability was determined by using single, low sensitive techniques.

In this work we were able to identify some of the genes that could be responsible for the differential cell viability levels found among 9 successive TMJF cell passages. Thus, our significance analysis of microarray data (SAM) showed that the expression of 5 genes linked to different pro or anti-apoptotic activities was significantly associated to average cell viability. On one hand, two apoptotic genes -TRAF5 and PHLDA1- [26] were inversely correlated with the average cell viability, and the expression of both genes was higher in cell passages with the lowest cell viability. Although functional genetic studies should be carried out to confirm this statement, these results may suggest that these genes could play a role on inducing apoptotic cell death in the passages with lower cell viability, and they could be responsible of the differential cell viability levels found among the different cell passages analyzed in this work. On the other hand, three apoptosis-inhibitor genes (SON, HTT and FAIM2) were inversely correlated with the average cell viability. In the first place, SON has been described as a gene involved in protecting cells from apoptosis. SON regulates the mitotic machinery, such as centrosome components and genes critical for microtubule dynamics, as well as the DNA repair machinery. Recent findings also predicted SON to be a master regulator of multiple cellular processes that depend on microtubules, including cell death [27]. In the second place, HTT may play a role in microtubule-mediated transport or vesicle function. Moreover, this gene could also be involved in signalling, transporting materials, binding proteins and other structures, and protecting against programmed cell death [28]. Similarly FAIM2 (Fas apoptotic inhibitory molecule 2) is able to protect cells from apoptosis [29] probably, when the average cell viability was high, these three apoptosis inhibitor genes were up-regulated in compensation and control of pro-apoptotic genes. This compensatory mechanism could create a life-death equilibrium along the 9 cell passages. However, the activation of pre and anti-apoptotic genes could also be explained by the presence of a mixed population of viable and non-viable cells.

Once we determined cell viability and cell proliferation on 9 sequential TMJF cell passages, we analyzed the function of these cells as putative fibrocartilage-forming cells. In this regard, it is important to determine the capability of these cells to synthesize and remodel the fibrocartilage ECM, including ECM fibrillar and non fibrillar components. At this point, we should reconsider that selection of an adequate cell passage of TMJF cells could be a key step to ensure the success of translational clinical approaches, not only from the standpoint of cell viability, but also from the functional point of view. However, numerous authors have reported that TMJ cells are prone to change their phenotype and often stop the synthesis of cartilage-specific molecules during culture and after sequential cell passaging [9], [16], [30]. For these reasons, the genetic changes that could take place along 9 consecutive cell passages of human TMJ disc cells still needs to be clarified. In the first place, our analysis revealed that expression of 15 ECM fibrillar components significantly decreased along all nine cell passages, although 68% of the genes did not significantly vary. It is noteworthy that some genes encoding for collagen I tended to decrease with subculturing as previously demonstrated by other authors [30]. Collagen I forms a major structural framework of the fibrocartilage ECM [31], and it is essential to maintain the biomechanical properties of this cartilage. For that reason, cells intended for future clinical use should express physiological amounts of these genes. Interestingly, although the highest coll I expression corresponded to the first 3 cell passages, the levels of coll I expression were high at all 9 cell passages analyzed here (>2.300 f.u.), suggesting that all passages could be able to express adequate coll I amounts. Another relevant ECM fibrillar component of the TMJ disc is coll II. Strikingly, the expression of this protein was low at all passages, which is in agreement with previous reports [31], and it did not vary along subculturing. Other less abundant collagen fibers such as coll III, IV, V, VI, IX, XII, XV and XVI also tended to decrease with sequential passaging. In general, these collagens form a 3-D structure that associates with coll I and II to constitute the main scaffold of the cartilage TMJ. Taking together, these results imply that most fibrillar ECM components did not vary with sequential culturing and, in the cases that tended to decrease, the expression levels are relatively high at most subcultures.

In the second place, the analysis of non-fibrillar ECM components confirmed that the majority of genes did not decrease along consecutive cell passaging, although some specific genes did significantly vary upon subculturing. Non-fibrillar components of the ECM play an important role in cartilage homeostasis, cell adhesion and hydrostatic balance. One of the most important non-fibrillar ECM components are glucosaminoglycans and mucopolysaccharides that tend to associate to proteins to form proteoglycans, which are able to attract water molecules via osmosis to keep the ECM hydrated, as well as growth factors [32]. In this regard, it is important to note that several relevant GAGs and PGs maintained their expression during sequential culturing including, versican, lumican, dermatan sulfate, etc. However, our results reveal that some genes could diminish their expression upon subculturing, including biglycan, decorin aggrecan and some genes encoding for chondroitin-sulfate, hyaluronic acid and heparan sulfate. This finding suggests that TMJF cells could not be able to generate an efficient fibrocartilage ECM at advanced cell passages. Chondroitin-sulfate may be the predominant proteoglycan present in cartilage. Interestingly, the highest intracellular sulfur concentrations correlated with the highest expression of chondroitin-sulfate genes, with the most functional levels found at P4 and P5. Hyaluronan synthase 1 (HAS1) plays a role in of hyaluronan/hyaluronic acid (HA) synthesis and may be involved in prevention of cartilage destruction by the continuous production of HA [33], [34]. However, the tendency to decrease that we found here reveals that these cultured cells should be used at the earliest cell passages.

On the other hand, biglycan, aggrecan and decorin are crucial components of the ECM. These proteins are involved in collagen fiber assembly and play an important role in the organization of the fibrillar ECM. Although the decreasing trend of these three proteins suggests that they should be used at the first cell passages, the gene expression levels of these elements were high at P5 as determined by microarray. Regarding other ECM proteins of interest, including glycoproteins our results showed that some cartilage-related genes decreased with culturing including chondrolectin, cartilage intermediate layer protein and cartilage oligomeric matrix protein, whereas two laminin genes tended to increase. Notwithstanding the role of these ECM components is less known, these results point out the need to use early cell passages in regenerative protocols. All our findings related to ECM components expression could suggest that TMJF cells could be functionally adequate until at least P5. The behavior from P5 to P9 could be associated to response mechanism to the de-differentiation effects caused by ex vivo adaptative conditions. In this context, is well known that the lack of cell-cell interaction and cell matrix interactions could play a major role in altering the gene expression of cultured chondrocytes [9] and the re-differentiation process is an attractive strategy for TMJF cells.

In summary, all these results allow us to suggest that determination of cell viability and functionality of human TMJF cell kept in culture using highly-sensitive methods must be one of the key parameters that should be determined during the quality control of TMJF for clinical cell transplantation, and we propose that all cells to be used for clinical purposes be previously analyzed using the highly-sensitive methods used in this work. In general, our data imply that the highest cell viability levels correspond to TMJF passages 6 and 5 and the most functional passage is the passage 5. We therefore suggest that cell passages P5 and P6 should be preferentially used in cell therapy and tissue engineering protocols using this cell type.
